# Comparative study of the effects of fetal bovine serum versus horse serum on growth and differentiation of primary equine bronchial fibroblasts

**DOI:** 10.1186/1746-6148-10-119

**Published:** 2014-05-26

**Authors:** Jana Franke, Vanessa Abs, Claudia Zizzadoro, Getu Abraham

**Affiliations:** 1 Institute of Pharmacology, Pharmacy and Toxicology, University of Leipzig, An den Tierkliniken 15, Leipzig 04103, Germany; 2 Division of Veterinary Pharmacology and Toxicology, Department of Veterinary Public Health, Faculty of Veterinary Medicine, University of Bari, Strada Prov.le per Casamassima, km 3, Valenzano, BA 70010, Italy

**Keywords:** Airways, Primary bronchial fibroblasts, Cell culture, In vitro, Serum types

## Abstract

**Background:**

Airway fibroblasts have become a critical addition to all facets of structural lung tissue changes such as in human asthma and chronic obstructive pulmonary disease, but little is known about their role in the equine recurrent airway obstruction, a disease that resembles to the human asthma. Since the equine bronchial fibroblasts (EBF) have not been isolated and characterized yet, the use of defined medium was investigated.

**Results:**

Primary EBF were cultured on non-collagen coated flasks without serum or in the presence of fetal bovine serum (FBS) or horse serum (HS) or in serum depleted medium. EBF cultured in serum-free basal media and those serum deprived were not able to proliferate and even exhibited considerable cell death. In media containing FBS or HS, proliferation of the cells was reproducible between different primary cultures and cells demonstrated expression of vimentin. Large variations were found in the ability of FBS and HS to support growth and differentiation of EBF in monolayer culture. Indications of growth-promoting actions, increasing passage number as well as maintaining fibroblast morphology were found rather in FBS than in HS. EBF culturing in HS needed longer doubling and confluence time. The protein content of the cell pellets was higher in EBF cultured in medium containing HS than FBS. Alpha-smooth muscle actin seemed to be less expressed in EBF cultured in medium containing FBS than those in HS.

**Conclusions:**

In sum, serum addition to basal EBF medium enhanced EBF differentiation into myofibroblasts, and these findings are useful to develop in vitro fibroblast culture models that mimic in vivo physiological processes and to study airway disease mechanisms and remodeling.

## Background

Chronic airway diseases like human bronchial asthma and chronic obstructive pulmonary disease (COPD) and the equine recurrent airway obstruction (RAO) principally characterized by bronchial hyperreactivity and airflow obstruction result from several factors including structural alterations of the airway wall and cell function. Such airway remodelling is a common feature of abnormal deposition of extracellular matrix (ECM) components in airway mesenchymal layer associated with airway wall thickness [1-3]. Airway fibroblasts and those cells differentiated into myofibroblasts contribute to sub-epithelial fibrosis linked to airway remodelling by producing ECM proteins such as collagen, fibronectin and proteoglycans [4,5].

There are several well characterized human fetal lung fibroblast cell lines that are cultured in defined media and for use as in vitro cell model to study airway diseases and remodeling, for example, HFL-1 [6], IMR-90 [7], HEL299, MCR-5 [8], WI-38 [9] and GM 06114 [10]. Culture of primary bronchial fibroblasts exists for human [11,12], mice [13] and rats [14]. However, there is currently neither description of equine adult primary airway fibroblast cultures nor there are such cell lines for this species.

Not all cells have the same requirements for growth and survival. With this regard, for successful growth, maintenance and expression of differentiated metabolic functions of human or animal cells *in vitro*, either primary cultures or continuous cell lines, appropriate culture conditions are required that mimic the physiological conditions *in vivo and situ*. In fact, it is well known that serum represents a fundamental source of nutrients, cytokines and adhesive molecules necessary for in vitro cell growth, metabolism and to stimulate proliferation [15]. In serum-free medium, only the addition of growth factors could initiate mouse lung fibroblast proliferation [16]. On the other hand, proliferating primary cardiac and dermal fibroblasts as well as established cell lines have been synchronized into a non-dividing G_0_-phase to cell cycle by serum withdrawal [17] which favour fibrotic processes or cause cell death [18,19]. While significant advances are made to culture conditions of permanent cell lines, current research is lacking that compares cultures of primary airway fibroblasts in different serum origins added to a standard culture medium or serum depleted medium, and how this may affect their differentiation. The sera that most widely used are bovine origin (adult or new born or fetal origin); as well the horse serum has been seen as an alternative to provide growth factors and hormones in modern cell biology. Indeed, almost it is not known about the responses of primary airway fibroblast cultures to horse serum factors.

Aim of the present study was to develop a primary bronchial fibroblast culture technique and investigate the influence of horse serum in comparison to FBS on cell viability, morphology and immunocytochemical characteristics, cell proliferation and α-smooth muscle actin expression (α-sma) to further understand the mechanism of peribronchial fibrosis (airway remodelling) in the equine RAO.

## Results

### Effect of serum absence and serum withdrawal on primary EBF culture

Primary EBF cultured without serum presence in basic DMEM failed to attach and to proliferate and those attached, disappeared completely from the flask surfaces within 1-week of culture. Also, we tested whether confluent EBF cultured in DMEM can be affected by serum withdrawal. Serum withdrawal led to modification of EBF number in culture; they exhibited detachment within three days of serum starvation (data not shown). Even the addition of 10% FBS after 24 h did not significantly enhance EBF attachment and proliferation.

### Cell viability and morphology under FBS and HS influence

**Figure 1:**
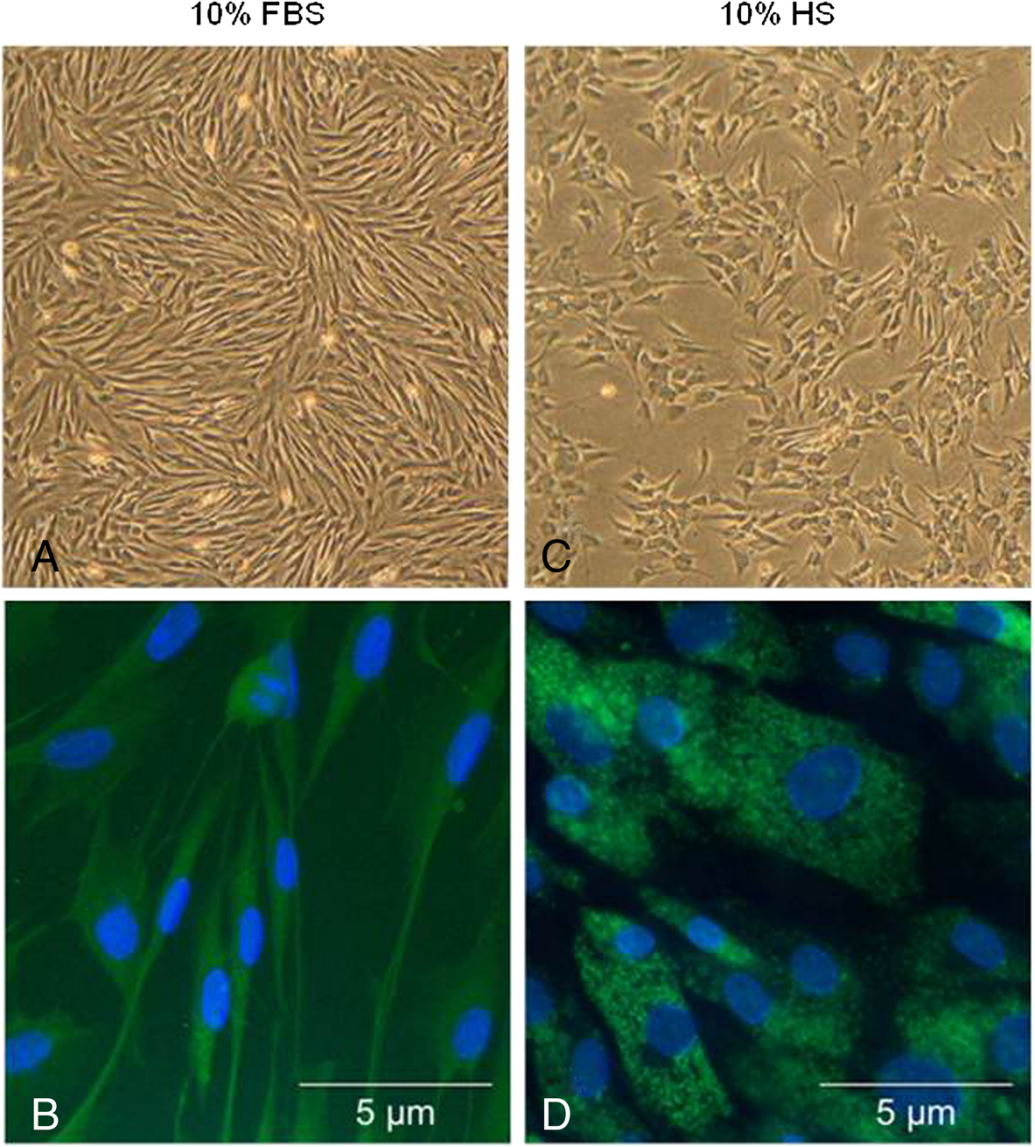
**Morphological and immunocytochemical appearance of EBF cultures in the presence of FBS or HS.** Phase contrast microscopy on day 3 of FBS culture **(A)** and HS culture **(C)***(Magnification: x40)*; Expression of Vimentin by EBF cultured in FBS **(B)** and HS **(D)***(Magnification: x400)*.

Cell yield from digested bronchial tissue was consistent. Under both culture conditions, EBF were stained with trypan blue and the percentage of viable cells was similar, usually > 95%. No significant evidence of cell necrosis or cell apoptosis was observed under inverted light microscopic analysis. With regard to cell morphology, EBF cultured in DMEM with 10% FBS appeared to be typically flattened and spindle-shaped (with a homogenous cytoplasm) over several weeks of passages compared to EBF cultured in DMEM with 10% HS (Figure 1A). In medium containing FBS, EBF were grown over the growth surface with only loose cell-cell-contact until reaching confluence and then formed tight parallel lines which remained as typical fibroblastic monolayer until passage 15. EBF between passages 16 – 20 started to change their morphology: cells were large, flat and more polygonal shaped, with a large, heterogeneous nucleus. At the same time, cell growth was rapidly reduced and cell viability was diminished. In contrast, EBF cultured in the presence of HS showed altered morphological changes within 2 days of culture; cells were small and more compact in shape combined with granula-like dark structures in the cytoplasm (Figure 1C). Moreover, cells grew in clusters and chains and failed to reach confluence within a week. This morphological behaviour of EBF was seen under this culture conditions until passage 7, but thereafter, EBF failed to proliferate regularly and decreased in their viability (passage 9). Under both culture conditions, most EBF (>99%) were positive for vimentin, but with more characteristic filamentous structures within the cytoplasm in medium containing FBS (Figure 1B) than in the presence of HS (Figure 1D).

### Sera effects on cell population, proliferation and total protein

**Figure 2:**
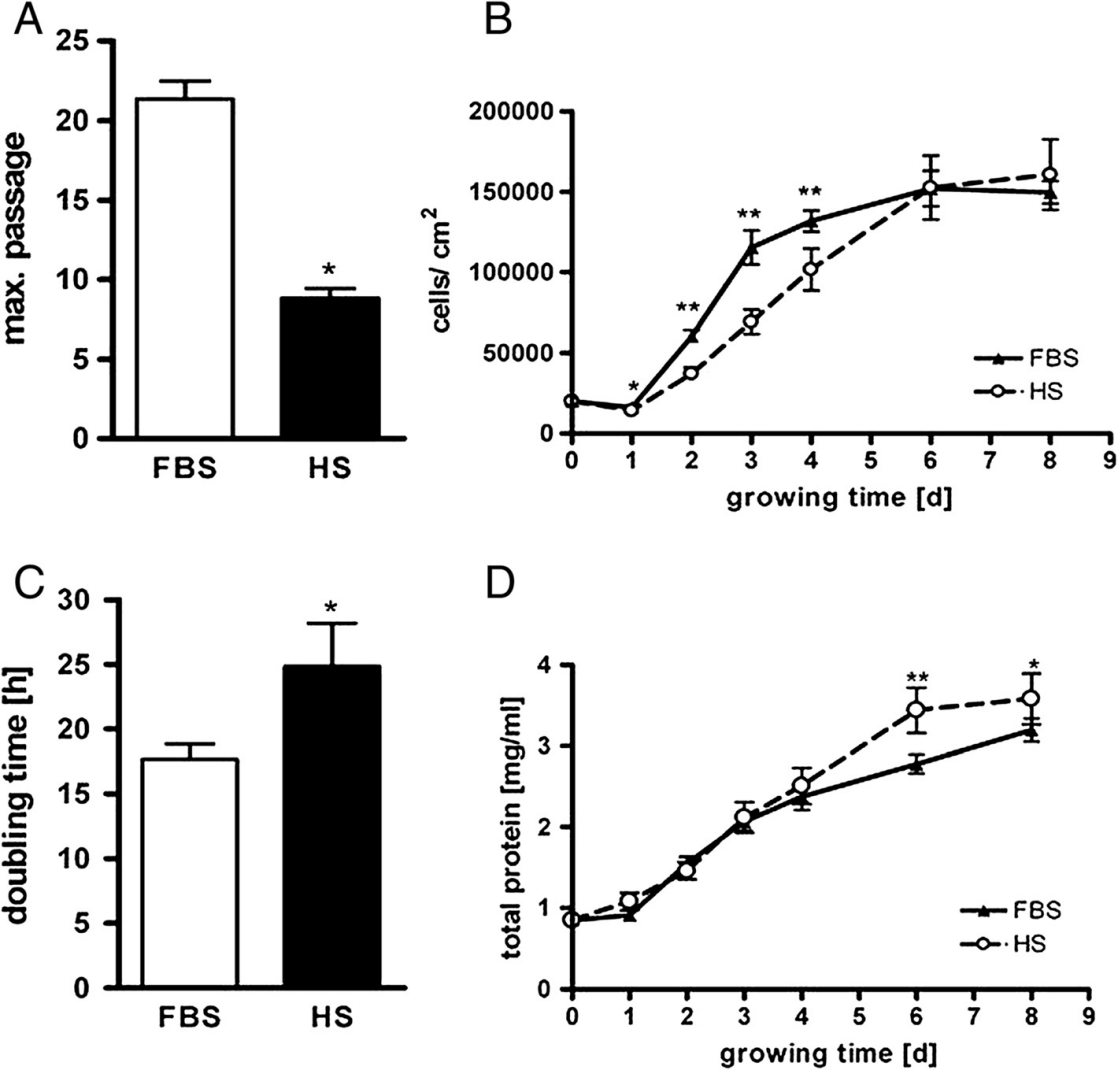
**Effect of FBS and HS on cell growth, doubling time, protein content and passage number.** EBF were passaged every week and seeded in a density of 2 × 10^4^ cells/cm^2^. EBF cultured in 10% FBS keep proliferating over a longer period than in 10% HS **(A)**. EBF were seeded in 6-well-plates at a density of 2 × 10^4^ cells/cm^2^. Effect of HS and FBS on growth curve **(B)**, population doubling takes longer in HS than in FBS (calculated between day 1 and day 3) **(C)** and effect of HS and FBS on total protein amount over 8 days **(D)**. Data are presented as means ± SEM. (*p < 0.05, **p < 0.01, n = 10).

Number of passages and proliferation differed significantly between the two culture conditions. By routinely passaging primary EBF (once a week), EBF maintained their typical morphological fibroblastic properties up to passage 20 in culture medium containing fetal bovine serum, whereas EBF in horse serum did exhibit significantly limited proliferation rate merely up to passage 9 (Figure 2A). Under both experimental conditions, growth curves of EBF showed similar initial 24 h lag-phase. The population data began to exhibit significant differences between the two conditions until day 4 of EBF culture (Figure 2B). Cell doubling time was slow in the presence of horse serum, and here, it took significantly longer than in culture medium containing FBS (Figure 2C). Under culture conditions in the presence of FBS, primary EBF reached confluence on day 4 but not in the presence of HS. Indeed, significant differences between serum groups were not found on day 6 and 8. Total protein content of the cell monolayers did not reveal significant difference between the two experimental conditions until day five (Figure 2D), while after day six the amount of total protein increased under HS condition and statistically differed from FBS condition, though cell number was the vice versa.

**Figure 3:**
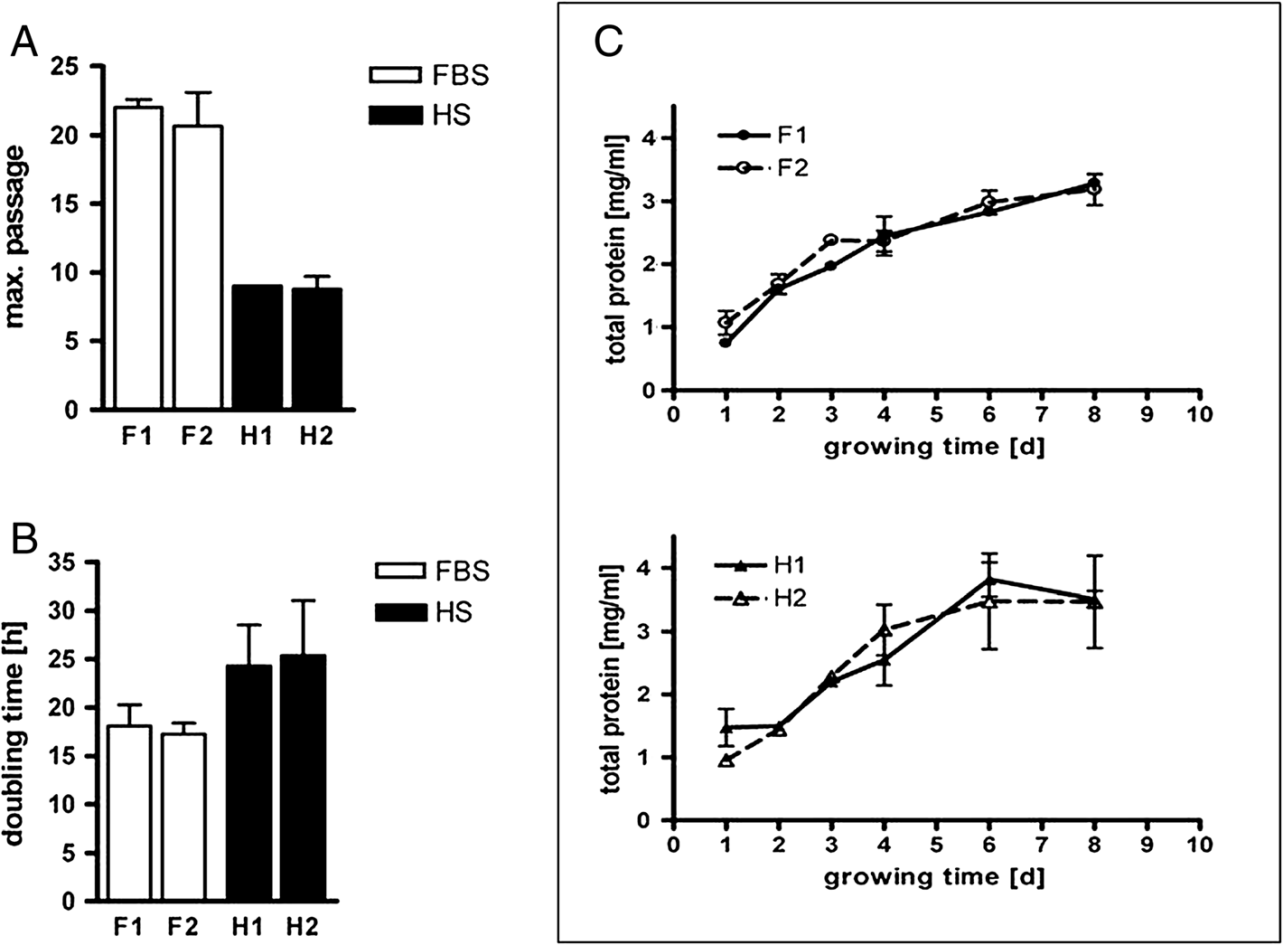
**Effect of FBS and HS batches on EBF passage number, population doubling and total protein content.** Under standard culture conditions as described in material and methods, cells were subjected to obtain **(A)** maximum passage number (n = 2), **(B)** population doubling (n = 5) and **(C)** total protein amount (n = 2). F1 and F2 represent FBS batches, and H1 and H2 the HS batches.

**Figure 4:**
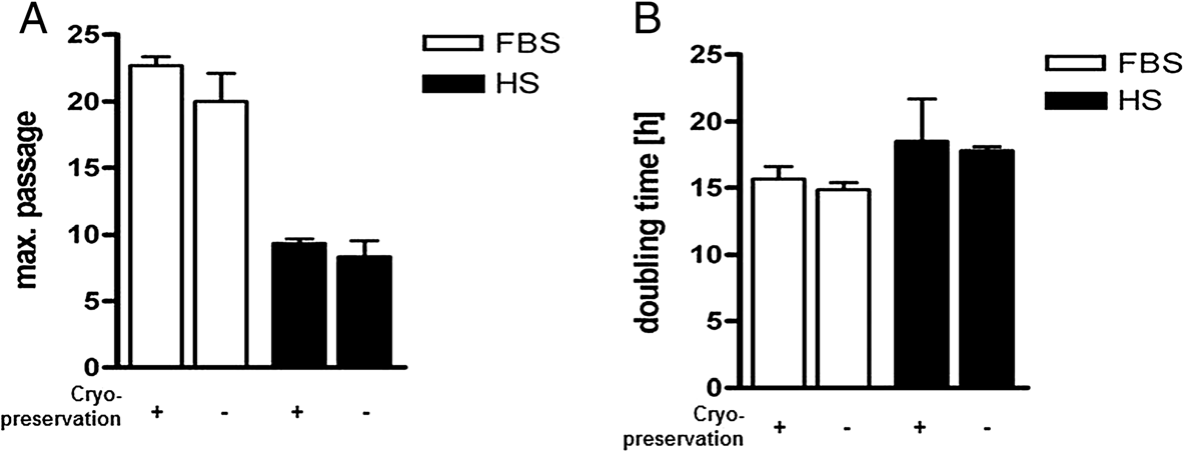
**Effect of cryopreservation on EBF passage number and population doubling.** Under standard culture conditions, cells were subjected to obtain **(A)** maximum passage number (n = 3) and **(B)** population doubling (n = 2). Thawed cells were used after 2 passages and treated as described under Figure 2.

When two different batches of FBS and HS were compared on maximum passage, population doubling time and total protein content of EBF, there was no remarkable differences between them (Figure 3). Moreover, cell proliferation was compared between freshly isolated and cryopreserved EBF. We did not observe any effect of cryopreservation on EBF proliferation (Figure 4); so routinely, thawed cells could be used for experimental purposes.

**Figure 5:**
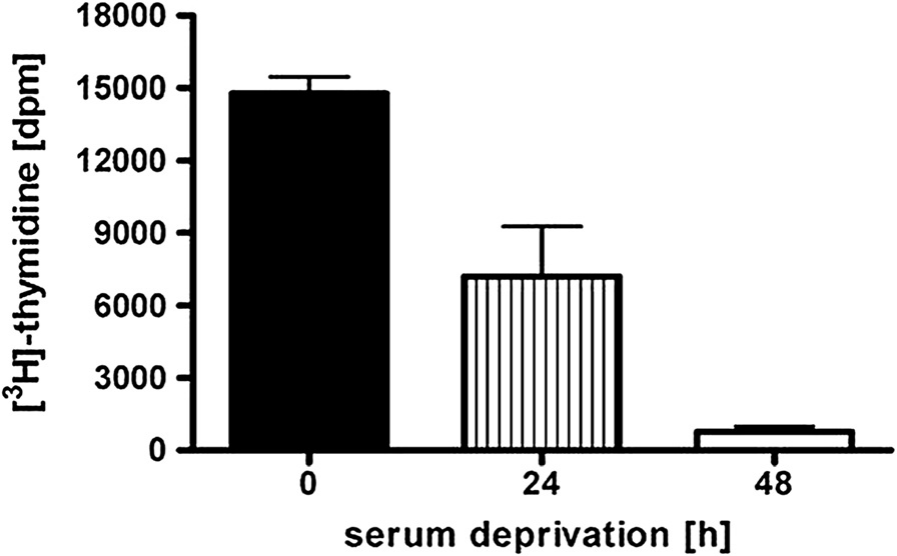
**Effect of serum-deprivation on [**^**3**^**H]-thymidine incorporation.** Sub-confluent EBF were subjected to serum deprivation for 24 and 48 hours. Cell proliferation was determined by [^3^H]-thymidine incorporation (n = 2).

Furthermore, we assessed the effect of time-dependent withdrawal of fetal bovine serum on cell proliferation in sub-confluent primary equine bronchial fibroblasts by [^3^H]-thymidine incorporation assay (Figure 5). 10% FBS induced markedly EBF proliferation compared to EBF cultured under serum withdrawal for 24 and 48 h. Even if this investigation was carried out in two to four cell isolates (n = 2) in triplicates, the results indicate that after 48 h of FBS deprivation, cells almost stopped to proliferate in comparison to 24 hours serum withdrawal.

### α-Smooth muscle actin expression

**Figure 6:**
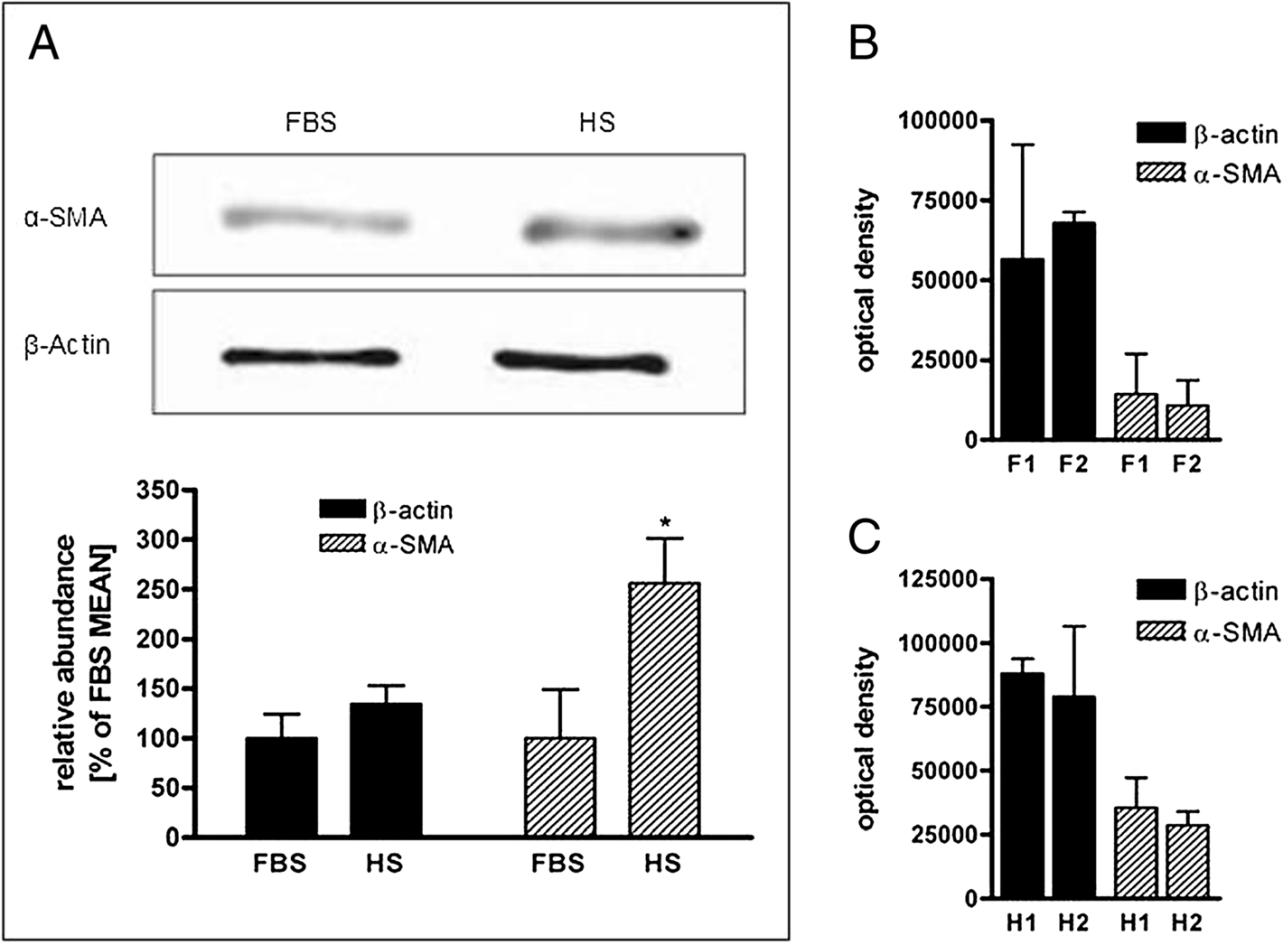
**Sera effects on β-actin and α-SMA expression.** Western blots were performed on whole cell lysate from EBF cultured for 4 days in DMEM containing 10% FBS or HS. Densitometry of β-actin and α-SMA was calculated by SynGene. **(A)** EBF cultured in HS show a higher α-SMA expression than EBF cultured in FBS (n = 5). Serum batch of **(B)** FBS and **(C)** HS (n = 2) did not affect α-SMA expression. F1 and F2 represent FBS batches, and H1 and H2 the HS batches. Data are presented as means ± SEM. (*p < 0.01).

To evaluate differentiation of fibroblasts into myofibroblasts, the expression of α-SMA was assessed in cells under culture conditions in the presence of FBS and HS. Under both experimental conditions, α-SMA was detectable in all cell lysates. In the presence of HS, α-SMA was significantly expressed in comparison to EBF cultured in medium containing FBS (Figure 6A). The expression of the control protein β-actin also differed between both culture conditions (Figure 6A); in the presence of HS, it tended to increase even if not statistically significant. Also, when two different batches of FBS and HS were used, and the effect on α-SMA and β-actin expression was compared, no significant difference was observed (Figure 6B, C).

## Discussion

The overall aim of the present investigation was to assess viability, morphological and proliferation differences of primary bronchial fibroblasts of the horse subjected to basal medium without serum, or first serum containing but later serum-depleted medium, in the presence of fetal bovine serum or horse serum. The results highlight differences in examined parameters when primary EBF were cultured within these differing conditions. In the absence of serum, basal medium DMEM did not support EBF attachment and proliferation; serum withdrawal in sub-confluent EBF has also led to decreased cell proliferation and attachment. The most important factors in significantly affecting cell growth and differentiation were the sera types added to basal DMEM. EBF cultures were established from freshly isolated EBF (P_0_) or from frozen isolated EBF. Despite cryopreservation, the proliferation data generated from frozen cells were similar to those from freshly solated and further cultured EBF.

After short-term trypsin digestion of peeled and minced equine bronchial mucosa and from cultures of epithelial cell mixture in DMEM containing FBS, we could observe a complete disappearance of epithelial cells within 1-week and instead five days later a complete coverage of culture flasks with viable primary equine bronchial fibroblasts. Thus, DMEM does not support epithelial cell growth despite the presence of serum. This isolation procedure is described, to our knowledge, the first time for these cells and resulted in reproducible large cell yields with typical mesenchymal cell properties. EBF cultured in serum-free DMEM were, however, not only unable to proliferate but also were non-adherent after seven days in culture. From this result, indeed, it is difficult to point out the proteins which are present in serum and absent in the basal medium that are essential for primary EBF growth. The reason why EBF disappeared seems to be, even if we did not proof, the occurrence of apoptosis and necrosis. Thus, it can be argued that under serum-free conditions in our cultures EBF-specific growth factors which are usually present in serum are missing. But in many established permanent cell line culture under serum-free conditions [20,21] the serum-free environment did support growth and differentiation with clonal growth as well as mass culture (for review see [22]).

Although recent efforts have shown that human mesenchymal stem cells and primary epithelial cells can be isolated and expanded long-term in serum-free medium [23-26], no published work had shown whether human or animal primary airway fibroblasts are able to expand under serum-free conditions for long-term in culture. The selective nature of serum-free media formulations could be used in primary cultures of tissue explants by allowing growth suppression of other cell types which might quantitatively influence experiments with primary cells obtained from tumor tissues. Presumably, in our case, the serum-free condition should be optimized for enhanced primary fibroblast expansion by adding growth factors which have to be further investigated. In sum, understanding of these processes is essential in understanding the use of these cells as in vitro model and to study their role in airway remodelling during respiratory disease mechanisms.

The importance of serum supplementation for cell attachment, growth and passaging of in vitro proliferating cells [27] as well as the influence of serum deprivation on permanent cell line proliferation and cycle [28] are well known. On the other hand, no data are available on the effect of time–dependent serum withdrawal on primary airway fibroblast proliferation and differentiation. Since serum components might affect cell stimulation, many studies in vitro are conducted in the absence of serum, and cells can be deprived of serum for 1 – 8 days [29,30], and thus, the aim of the present study was to investigate the response to serum withdrawal for 24 to 48 hours in sub-confluent primary equine bronchial fibroblasts which were first cultured in DMEM containing 10% FBS. In our study, we have clearly demonstrated a marked time-dependent decrease in [^3^H]-thymidine incorporation in cells deprived of serum for 24 to 48 hours, suggesting discontinuation of proliferation in the absence of FBS. After 72 hours of serum depletion many cells were floating in the medium and the number of attached cells was decreasing, indicating altering cell viability and morphology, in agreement with data found for cardiac fibroblasts [18]. These findings show that EBF in primary cultures are not able to adapt to serum deprivation for a given time, presumably sufficient serum growth factors are not available.

Moreover, we have tested the effects of sera types on proliferation, morphology, population doubling and viability of primary equine bronchial fibroblasts. The results highlight striking differences in cell proliferation, morphology, passaging time and number as well as total protein amount and α-SMA expression when EBF were cultured within these differing conditions. Both FBS and HS are natural products, presumably, with varying concentrations of growth factors within different batches; however, the effects measured in our study were not dependent on batch/lot number. EBF grown in DMEM containing 10% FBS continue to proliferate even after reaching confluence, whereas these same cells, when cultured in DMEM containing 10% HS, had relatively limited proliferation rate, longer population doubling time and somehow quite different morphological features. Cells in HS were smaller, chain-forming and more compact in shape with dark cytoplasmic granules which can be related to the occurrence of abnormal protein accumulation than those in FBS, and they failed to reach confluence. In concordance, sera types affect other various cell types in a similar way: bovine adipocytes and sheep skeletal muscle satellite cells proliferated rapidly, when FBS was supplemented to growth medium instead of HS; thus, FBS-containing medium is often used as growth medium in these cultures [31,32]. On the contrary, equine chondrocytes could equally proliferate under both conditions [33]. Even if they are equine cells, it is noteworthy to find that EBF exhibit a preference for fetal bovine serum factors over the horse serum factors, whereas the vice versa has been seen for neural cells [34]. Merely, early studies have proposed that horse serum is appropriate for neuronal cell cultures [34,35].

Moreover, despite the short passaging time and limited proliferation and population doubling rate, increased α-SMA expression was accompanied by higher protein amount in the presence of horse serum than in bovine serum, suggesting that HS might stimulate fibroblast differentiation into myofibroblasts. It is well known that myofibroblasts are able to produce large amount of extracellular matrix (ECM) proteins, as well as growth factors and cytokines and express cytoplasmic contractile structures including α-SMA. Thus, it seems that the horse serum increases the protein content in EBF by increasing synthesis of structural proteins, ECM and cytokines. Indeed, it is not yet known if differential expression of α-SMA and protein is functionally relevant.

## Conclusions

Fetal bovine serum favors fibroblastic morphology with enhanced proliferation rate, population doubling, passage number and triggers cell differentiation (as vimentin staining showed), suggesting serum factors essential for the equine airway fibroblasts are available in the fetal bovine serum, whereas in the horse serum cells there were signs of degeneration or cell granularity. Moreover, we can conclude that serum withdrawal for 48 hours rather decrease EBF adaptation and enhance cell detachment than the 24 hour FBS depletion and in the latter case cell viability was though not altered, thus, EBF cultured in 10% FBS represent a good model allowing studying the response to drugs that influence cell proliferation and pathways of airway remodeling in airway diseases.

Study limitation: Animal serum is a complex mixture of a large number and variety of components; therefore, it is difficult to assess, at this stage, the significance of the observation that horse serum inhibits the programmed progress of equine airway cells through the lineage of differentiation in cultures.

## Methods

### Isolation and culture of equine bronchial fibroblasts

Primary equine bronchial fibroblasts were cultured in Dulbecco’s Modified Eagle Medium (DMEM) supplemented with 200 Units/ml Penicillin, 0.2 mg/ml Streptomycin, 5 mg/ml Amphotericin B (PAA Laboratories GmbH, Pasching, Austria). Surfaces of culture flask were not pretreated with adhesion supporting matrix like collagen.

Bronchial segment tissue samples were obtained from adult non-diseased slaughter horses of different breed, age and sex and slaughtered at local abattoirs (Freiberg, Germany). Primary equine bronchial fibroblasts (EBF) were isolated from these tissues. Briefly, the bronchial mucosa was removed from the bronchi, washed, minced to about 1-3 mm pieces, and 500 mg tissue were digested with 0.25% trypsin/EDTA (Sigma Aldrich, Deisehnhofen, Germany) in Hanks’ balanced salt solution (HBSS) (PAA Laboratories GmbH, Pasching, Austria) in 50-ml Erlenmeyer glass flasks and incubated at 37°C for 2 hours in humidified atmosphere of 5% CO_2_. Trypsinized samples were then filtered through sterile double-layered gauze and rinsed twice with ice-cold HBSS. Cell suspension was then further sieved through sterile nylon cell strainers (mesh size: 40 μm) (BD Biosciences, Franklin Lakes, NJ) and centrifuged two times to remove tissue debris.

To evaluate the effect of sera on proliferation and differentiation of primary equine bronchial fibroblasts, we used at least two to three different lots of cell culture appropriate not inactivated fetal bovine serum (FBS) (Gibco, Carlsbad, CA, USA) and horse serum (HS) (Sigma Aldrich, Deisehnhofen, Germany). Since it was not the objective of the study, we did not compare different serum products of several companies. FBS is routinely used to culture other cell lines in our laboratory, and should be tested before use; thus, after cell isolation, final cell pellets were first resuspended and cultured in DMEM containing 10% fetal bovine serum (FBS) for at least up to two passages. Thereafter, EBF were trypsinized and sub-cultured under different conditions: a) in DMEM without serum, or b) first in DMEM containing FBS and then serum deprived, or c) in DMEM in the presence of 10% FBS or d) in DMEM in the presence of 10% horse serum (HS). Cells were routinely passaged every 7 days with replacement of medium every 2 to 3 days. Unless and otherwise specified; cells were routinely cultured in 75 cm^2^ flasks (Greiner Bio-One, Frickenhausen, Germany).

### Cryopreservation and thawing of EBF

Certain density of isolated primary EBF were either provided for direct culturing or frozen in 1 ml DMEM containing 20% FBS, 10% DMSO (Sigma Aldrich, Deisenhofen, Germany), penicillin, streptomycin and amphotericin B in liquid nitrogen until use. Also, some passages of EBF cultures were frozen. Cells were quickly thawed at 37°C in water bath and subsequently transferred into culture flasks with tempered culture medium (see above). Thawed cells were used for our study after 2 passages.

### Cell viability, morphology and immunostaining

For cell counting and viability testing, cells were trypsinized and washed with PBS and stained with trypan blue dye (Sigma-Aldrich, Deisenhofen, Germany). Cells excluding the dye were counted using Neubauer cell chamber. Also, after 1-week of EBF culture in two conditions, i.e., DMEM + FBS or DMEM + HS, fibroblasts were trypsinized, centrifuged and an aliquot was re-suspended in PBS with trypan blue.

EBF were plated into 75 cm^2^ flasks at a density of 2 × 10^4^ cells/cm^2^ and grown in 10 ml defined DMEM in the presence of 10% FBS or HS. Cell morphology was evaluated under inverted light microscope (Olympus CKX41). Digital images were taken from cells at different days and at possible confluence.

Expression of intermediate filaments in primary cultured EBF was evaluated using immunofluorescence staining against vimentin using a primary mouse monoclonal antibody and a secondary anti-mouse FITC-antibody (Dako Deutschland GmbH, Hamburg, Germany). EBF were cultured under the indicated culture conditions on glass cover slips (Carl Roth GmbH, Karlsruhe, Germany), washed twice with PBS after medium removal, and fixed in ice-cold acetone for 5 min at -20°C and immunostained as previously described [36].

### Cell proliferation assay and population doubling time

To further assess the effects of sera types on cell proliferation and population doubling, EBF between passage 3 and 6 were transferred into 6-well plates (2 × 10^6^ cells/cm^2^) and cell numbers were determined manually after day 1, 2, 3, 4, 6 and 8. Each time, the average from 2 wells was taken. The mean cell number was logarithmically transformed and the linear regression slope was calculated to derive the doubling time (DT). Proliferation rate was also determined in cryopreserved, thawed and cultured cells.

### [^3^H]-thymidine assay

Furthermore, cell proliferation was measured by [^3^H]-thymidine incorporation assay [11]. EBF (40 000 – 60 000 cells/well) were seeded into 12-well plates, in DMEM in the presence of 10% FBS, grown until 60% confluence, and further cultured in the presence of 10% FBS or serum-withdrawn for 24 or 48 hours. In all three settings, [^3^H]-thymidine (37 kBq/well) (Perkin Elmer, Waltham, MA) was added to the culture medium. 24 hours after incubation of cells with [^3^H]-thymidine, cells were washed in ice-cold PBS and incorporated radioactivity was determined by liquid scintillation counting (Beckman LS 6500 Scintillation Counter). Also, in thawed and cultured EBF, proliferation experiments were performed about 1-week after thawing, to avoid a major increase in the number of cumulative population doublings compared to experiments done with fresh cells.

### Cell growth determination

To measure the effect of sera on cellular growth, total protein was measured at defined time points during culture in 6-well plates by the colorimetric method [37]. In brief, EBF were rinsed twice in PBS and then trypsinized, centrifuged at 500 × g for 10 min. After removal of the supernatant, cell pellets were resuspended in PBS and sonicated 4 rounds for 30 second. Crude cell lysates were then diluted in 0.1 M potassium phosphate buffer (pH 7.4), incubated with copper in alkaline solution for 10 minutes at room temperature. After addition of Folin (Merck, Darmstadt, Germany), reduction of copper was completed within 45 minutes and quantitative analysis could be carried out by spectrophotometry at 660 nm (Beckman DU640 spectrophotometer, Beckman Coulter, Krefeld, Germany).

### Western blot

Primary EBF grown in 6-well plates under different culture conditions were harvested and suspended in sample buffer (containing 2% SDS, 25% [v/v] glycerol, 60 mM Tris–HCl and 0.1% bromphenol blue, 14.4 mM β-mercaptoethanol, pH 6.8) and boiled for 5 min. Equal amounts of whole cell lysate (2,5 × 10^5^ cells/ml) were subjected to SDS-PAGE; samples were separated on 12% acrylamide gel under reducing conditions and transferred to a nitrocellulose membrane (Whatman GmbH, Dassel, Germany). The membrane was then blocked in 3% BSA (PAA Laboratories) with TBST (20 mM Tris–HCl, pH 7.5, 150 mM NaCl and 0.05% [v/v] Tween-20) for 1 h at room temperature. α-SMA was detected following an overnight incubation of samples with mouse monoclonal antibody against human anti-α-SMA (1:1000; Sigma-Aldrich) and β-actin was detected using mouse monoclonal antibody against human anti-β-actin (1:10,000; Sigma-Aldrich) in 3% BSA-TBST at 4°C. Band visualisation was performed using alkaline phosphatase conjugated secondary anti-mouse igG antibody (1:5000; Promega GmbH, Mannheim, Germany) over 1 h at room temperature. Enzyme activity was detected using Western Blue stabilised substrate for alkaline phosphate (Promega). The membranes were digitalized and quantitated by densitometry analysis (SynGene, Cambridge, UK).

### Data and statistical analysis

All data are expressed as means ± SEM. Statistical significance of differences was evaluated by paired two-tailed student’s *t*-test using GraphPad Prism version 5.1 (GraphPad Software, San Diego, CA, USA). P < 0.05 was considered significant.

## Competing interests

All authors declare that no Competing interests exist.

## Authors’ contributions

JF, VA, CZ and GA designed the research and wrote the paper. JF and VA performed the experiment and analyzed the data. JF analyzed the data and wrote the paper. GA analyzed the data, wrote and approved the paper. All authors read and approved the final manuscript.
